# Two subtypes of surgery-categorized upper-lateral intracavitary pregnancy identified by MRI, a retrospective study

**DOI:** 10.1186/s12884-022-05274-x

**Published:** 2022-12-08

**Authors:** Weili Xie, Huan Yang, Shuo Shao, Ning Zheng

**Affiliations:** 1 School of Clinical Medicine, Jining Medical University. Forty-five South Jianshe Road, Jining, 272013 Shandong China; 2 Department of Radiology, Jining No. 1 People’s Hospital. Six Jiankang Road, Jining, 272011 Shandong China

**Keywords:** Angular pregnancy, Pregnancy outcome, Magnetic resonance imaging, Ultrasonography, Gestational sac

## Abstract

**Background:**

The pregnancy outcomes in women with surgery-categorized upper-lateral intracavitary pregnancy (ULIP), previously named angular pregnancy, demonstrate higher heterogeneity than in women with ultrasonography-categorized ULIP. We aimed to use preoperative MRI and correlated clinical characteristics to explore whether the surgery-categorized ULIP comprises obstetric conditions undefined by the current ultrasonography-based diagnostic criteria.

**Methods:**

This retrospective study involved 28 women with surgically and pathologically confirmed ULIP from January 2016 to July 2022. Two board-certified radiologists, blinded to the patients’ information, independently reviewed the MRI images, and determined each MRI feature, including endometrial thickness (EMT) and peri-gestational sac (GS) endometrial interruption. Disagreements were resolved by discussion to achieve a consensus. Based on the cutoff value of EMT (11.5 mm), the patients were divided into above-cutoff EMT (*n* = 22) and below-cutoff EMT (*n* = 6) groups.

**Results:**

Two subtypes of surgery-categorized ULIP were identified. Type-I ULIP (*n* = 22; EMT ≥ 11.5 mm), when compared to the type-II ULIP (*n* = 6; EMT < 11.5 mm), demonstrated lower incidence of peri-GS endometrial interruption (2/22 [9.1%] vs 6/6 [100%]; *P* = 0.001), higher logarithmic ß-human chorionic gonadotropin (ß-hCG) concentration (4.7 ± 0.4 mIU/ml vs 4.2 ± 0.6 mIU/ml; *P* = 0.026), lower rate of repeated dilatation and curettage (1/22 [4.6%] vs 4/6 [66.7%]; *P* = 0.003), less intraoperative blood loss (10.1 ± 6.3 ml vs 28.3 ± 18.3 ml; *P* = 0.001), and shorter hospital stay (2.8 ± 1.7 days vs 7.5 ± 3.8 days; *P* = 0.001). The peri-GS endometrial interruption negatively correlated with EMT (Odds ratio [OR] = 0.55; *P* = 0.001) and logarithmic ß-hCG concentration (OR = 0.08; *P* = 0.045). The below-cutoff EMT negatively correlated with ß-hCG concentration (OR = 0.06; *P* = 0.021).

**Conclusions:**

Surgery-categorized ULIP comprised two obstetric conditions among which the type-II ULIP, possessing unique imaging features undocumented in the literature, requires further attention during clinical practice.

## Background

Upper-lateral intracavitary pregnancy (ULIP), previously named angular pregnancy, is a type of potentially viable, eccentric, intracavitary pregnancy located in the cornua of the normal uterus [1]. In 1981, Jansen and Elliot proposed the surgery-based diagnostic criteria for ULIP with a key sign of eccentrically distended uterus accompanied by lateral displacement of the round ligament. By 2014, eighty-five cases of surgery-categorized ULIP have been reported and the pregnancy outcomes are highly heterogeneous [2]. The rates of live birth, spontaneous abortion, elective abortion, uterine rupture, abnormal placentation, hysterectomy, and maternal death are 21, 15, 24, 24, 9, 22, and 4% respectively [2]. Using ultrasonography, Grant A. et al. identify a complete peri-gestational sac (GS) endometrium as a key feature of ULIP [3]. The complete peri-GS endometrium was later adopted by Bollig and Schust in their case series study in which they report that the rates of live birth and early pregnancy loss are 80 and 20% respectively [4]. The promising results of Bollig and Schust’s study appear to encourage expectant management, whereas the high proportion of unfavorable outcomes in surgery-categorized ULIP remains a concern. The distinct pregnancy outcomes between surgery-categorized and ultrasonography-categorized ULIP raise a dilemma in the decision-making between expectant management and deliberate termination, especially in developing countries where the rates of induced abortion and unsafe abortion are unevenly high [5, 6]. Thus, developing noninvasive, preoperative diagnostic criteria for ULIP is of great importance.

While ultrasonography is widely used as the first-line screening test for pregnancies, the higher heterogeneity of the pregnancy outcomes shown in the surgery-categorized ULIP suggests that the current ultrasonography-based diagnostic criteria for ULIP may have missed some obstetric conditions in women with surgery-categorized ULIP, especially those with unfavorable outcomes.

With superior soft-tissue contrast, MRI may provide detailed imaging information regarding the intrauterine anatomical and physiological aspects in women with surgery-categorized ULIP [7, 8]. In the present study, we aimed to determine whether the surgery-categorized ULIP comprises obstetric conditions with differentiable preoperative MRI features that haven’t been addressed by the ultrasonographic diagnostic criteria.

## Methods

### Study patients

**Fig. 1:**
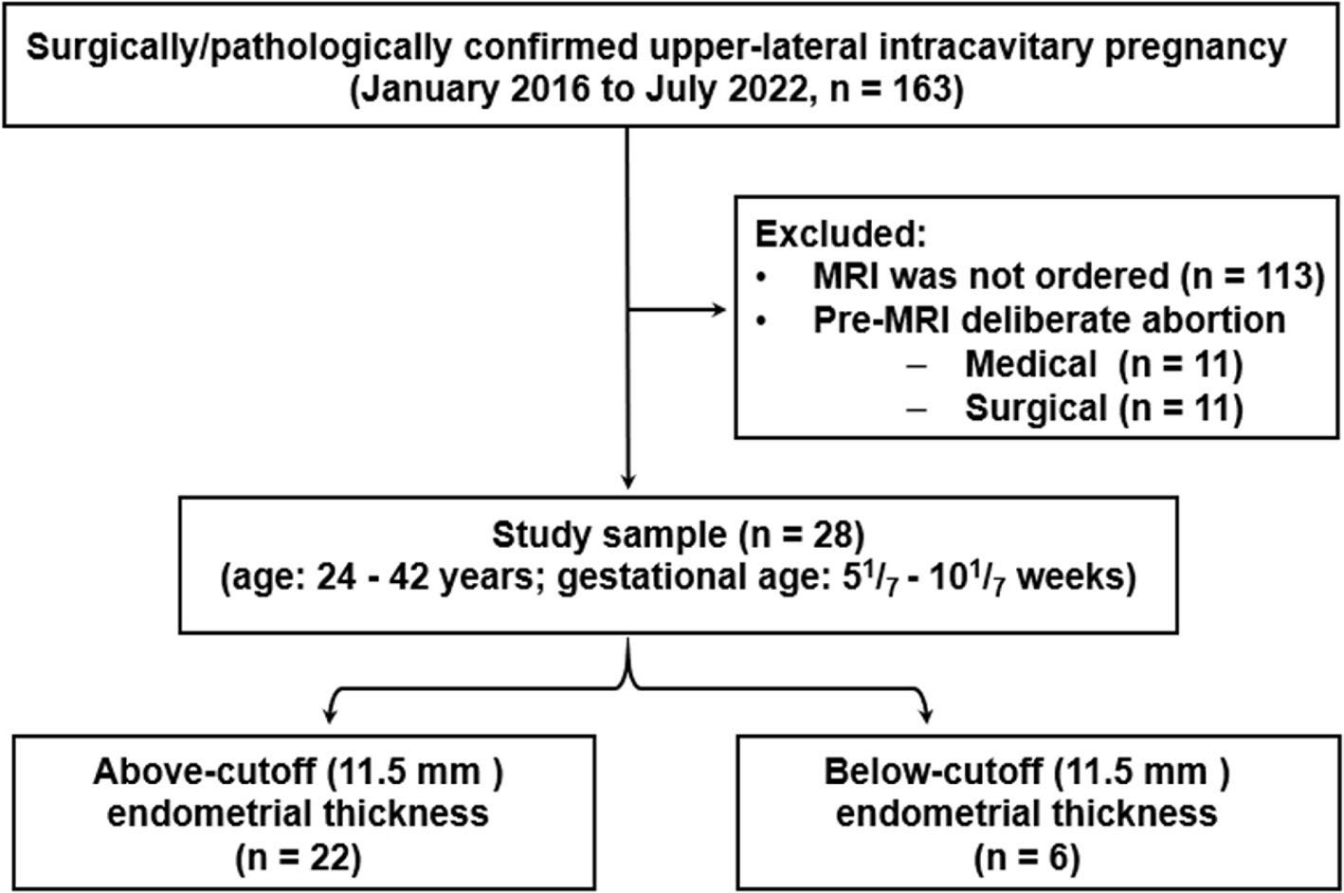
Flow chart of the study design

Our institutional review board approved this retrospective study and waived the requirement for written informed consent. Shown in fig. 1, we used our clinical database to consecutively review the medical records of the women who voluntarily chose surgical abortions and were confirmed with upper-lateral pregnancies (*n* = 163) between January 2016 to July 2022. The exclusion criteria were as follows: (a) MRI was not ordered (*n* = 113); and (b) received pre-MRI medical (*n* = 11) or surgical (*n* = 11) abortions. The study sample comprised 28 patients (age: 24–42 years; gestational age: 5^1^/_7_–10^1^/_7_ weeks). Based on the endometrial thickness (EMT), we divide the patients into two groups, the above-cutoff EMT group (*n* = 22), and the below-cutoff EMT group (*n* = 6). We used 11.5 mm as the cutoff value of EMT owing to the following reasons. First, EMT is an objective MRI measurement. Second, we have reported that the cutoff value of 11.5 mm is the imaging feature to differentiate interstitial pregnancy, a clinical entity inevitably proceeding to an unfavorable pregnancy outcome [7]. Third, we assumed that the surgery-categorized ULIP comprised different obstetric conditions potentially bifurcating into either favorable or unfavorable outcomes so that we could use the previously reported interstitial pregnancy, with a below-cutoff EMT [7], as a positive control.

### MRI protocol

In the present study, MRI was conducted on an Ingenia 3-T (Philips Healthcare) using a 16-channel anterior phased array coil plus 12-channel built-in table coils. Axial, coronal, and sagittal images were collected. Each plane has 24 slices. Details were explained in Table 1.

**Table 1:** MRI Parameters

MRI Parameters	Axial T1WI	Axial T2WI fat-suppress	Sagittal T2WI fat-suppress	Coronal T2WI
**TR (ms)**	400–600	2500–5000	3000–5000	1700–5000
**TE (ms)**	14	100	100	130
**FOV (mm**^**2**^**)**	240 × 351	300 × 300	250 × 250	260 × 350
**Voxel Size (mm**^**3**^**)**	0.9 × 1.0 × 5.0	0.9 × 0.9 × 5.0	0.8 × 0.8 × 4.0	0.8 × 1.0 × 5.0
**Gap (mm)**	1	1	1	1
**Acquisition time (s)**	115	192	201	98
**TSE factor**	4	27	28	22
**NSA**	1.6	1	1	2

TR: Repetition time

TE: Echo time

FOV: Field of view

NSA: Number of signal averages

### Image analysis

All images were reviewed independently by two board-certified radiologists with an experience of 20 and 15 years, respectively. Both MRI reviewers were blinded to the patient’s medical history, surgical findings, and pathological reports. Any disagreement was resolved by discussion to achieve a consensus.

**Fig. 2:**
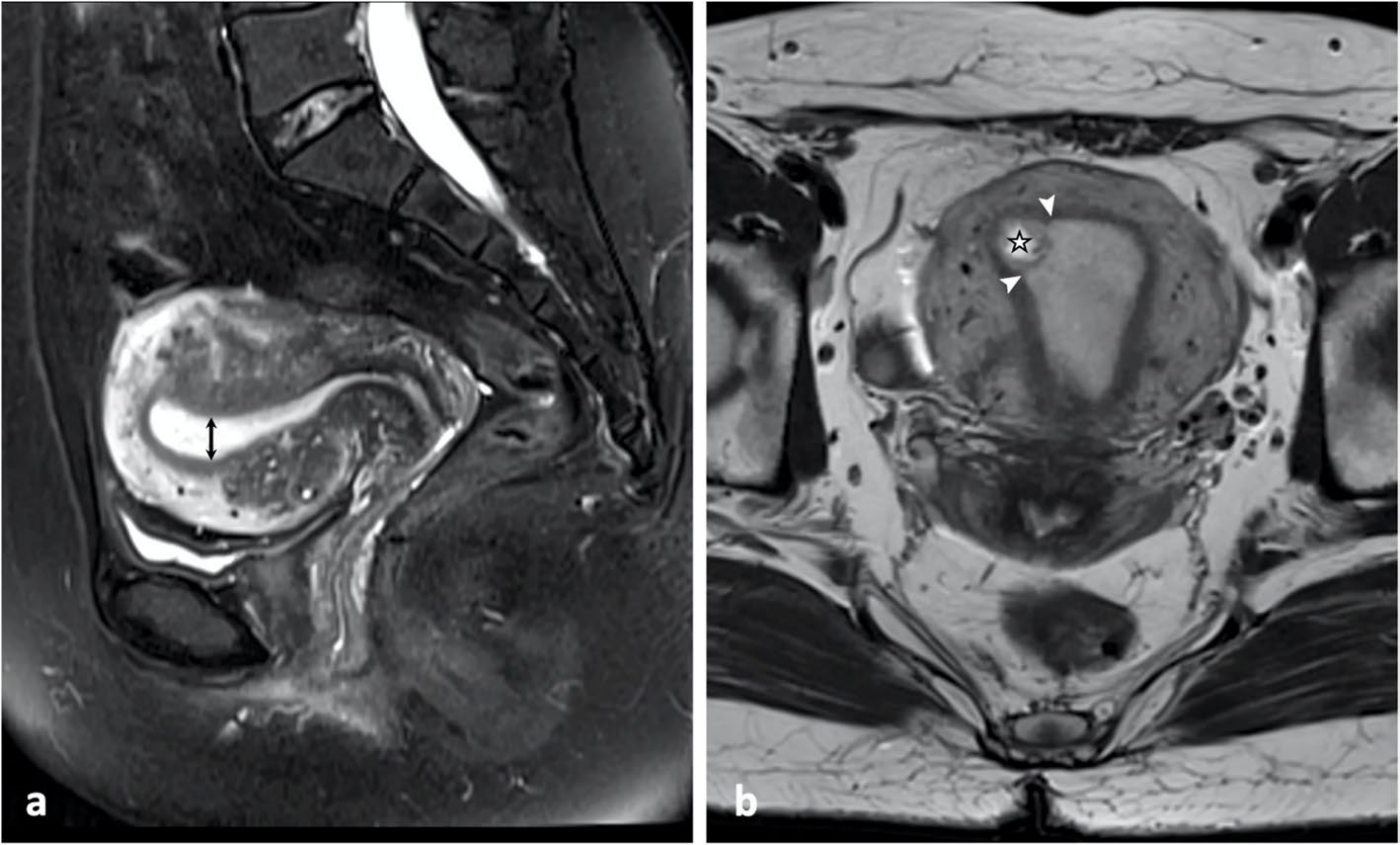
MRI in a 32-year-old patient with below-cutoff (11.5 mm) endometrial thickness. (a) Uterine sagittal fat-suppressed, T2-weighted imaging showed an endometrial thickness (double-ended arrow) of 8.6 mm. (b) Uterine coronal T2-weighted imaging showed a gestational sac (star) and interrupted peri-gestational sac endometrium (arrow heads)

**Fig. 3:**
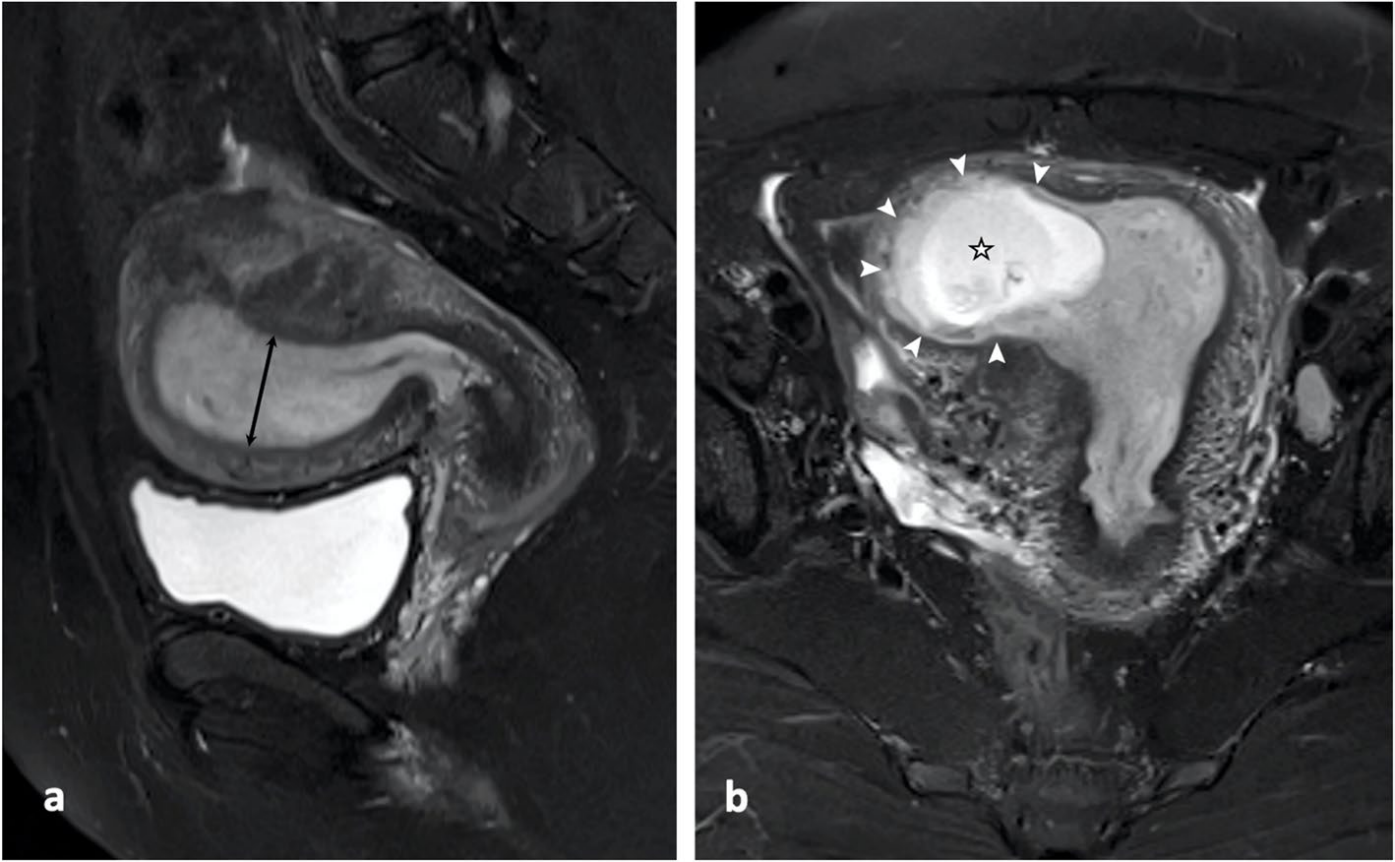
MRI in a 29-year-old patient with above-cutoff (11.5 mm) endometrial thickness. (a) Uterine sagittal fat-suppressed, T2-weighted imaging showed an endometrial thickness (double-ended arrow) of 22.6 mm. (b) Uterine coronal fat-suppressed, T2-weighted imaging showed a gestational sac (star) and continuous peri-gestational sac endometrium (arrow heads)

The MRI review focused on the anatomical and physiological relationships between the gestational sac and surrounding soft tissue. Two MRI characteristics were assessed, EMT [7] and the aspect of peri-GS endometrium. EMT was measured in fat-suppressed, T2-weighted sagittal images. We used 11.5 mm as the cutoff value, which has been previously reported [7]. A peri-GS endometrial interruption, shown in T2-weight uterine coronal image, was characterized as the discontinuity of the endometrium surrounding the GS (Fig. 2). Continuous per-GS endometrium was defined as the uninterrupted endometrium surrounding the GS (Fig. 3).

### Reference standard

Upper-lateral intracavitary pregnancy was confirmed by direct intraoperative observation of an eccentrically distended uterus accompanied by lateral displacement of the round ligament. The presence of gestational tissue was confirmed by the microscopic view of chorionic villi and/or an extravillous trophoblast.

### Statistical analysis

Data were analyzed using SPSS 26.0 software (IBM Inc.). Fisher’s exact test was used to analyze categorical variables including the presence or absence of vaginal bleeding, abdominal pain, dilatation and curettage (D&C), and peri-GS endometrial interruption. Numerical data, including the patient’s age, gestational age, sagittal EMT, and ß-human chorionic gonadotropin (ß-hCG) concentration were assessed for normality distribution and homogeneous variance by the Shapiro-Wilk test and Levene’s test, respectively. As all the data sets met assumptions of homogeneity of variance and normality, we used the Student’s *t* test to compare the mean of each parameter between the below-cutoff EMT and above-cutoff EMT groups. We used one-way ANOVA followed by Dunnett’s post hoc test to compare the EMT among the patients receiving either zero, one, or more than two times of D&C, and to generate the figures (Prism9, GraphPad Software Inc.). The simple logistic regressions were conducted to determine the correlations between MRI and clinical characteristics. A *P* value less than 0.05 was considered statistically significant.

## Results

### MRI and clinical characteristics of the patients.

**Fig. 4:**
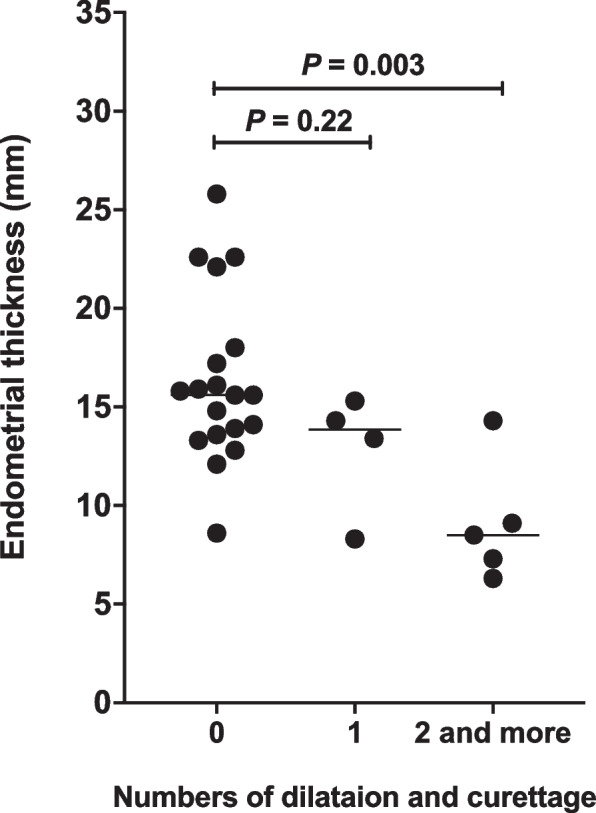
Endometrial thickness in women received either zero, one, or more than two times of dilatation and curettage prior to the current pregnancy. One-way ANOVA followed by Dunnett’s post hoc test was used to determine the means of women received zero (16.3 ± 4.3 mm; 95%CI: 14.3, 18.2), one (12.8 ± 3.1 mm; 95% CI: 9.8, 15.8), or more than two times (9.1 ± 3.1 mm; 95%CI: 5.2, 13) of dilatation and curettage

Table 2 summarized the MRI and clinical characteristics of the women with either below-cutoff or above-cutoff EMT. The incidence of peri-GS endometrial interruption (Fig. 2) in below-cutoff endometrium group (6 of 6, 100%) was higher than in above-cutoff endometrium group (2 of 22, 9.1%; *P* = 0.001). Ninety-one percent of the women with above-cutoff EMT (20 of 22) showed continuous peri-GS endometrium (Fig. 3). The mean of logarithmic ß-hCG concentration in below-cutoff endometrium group (4.2 ± 0.6 mIU/ml; 95% confidence interval [95% CI]: 3.7, 4.8) was lower than in above-cutoff endometrium group (4.7 ± 0.4 mIU/ml; 95% CI: 4.5, 4.8; *P* = 0.026). Women with above-cutoff EMT demonstrated less intraoperative blood loss (10.1 ± 6.3 ml vs 28.3 ± 18.3 ml; *P* = 0.001) and shorter hospital stay (2.8 ± 1.7 days vs 7.5 ± 3.8 days; *P* = 0.001) than women with below-cutoff EMT. The rate of repeated D&C (≥ 2 times) in women with thinner endometrium (4 of 6; 66.7%) was higher than in women with thicker endometrium (1 of 22; 4.6%; *P* = 0.003). No differences were identified in below-cutoff versus above-cutoff endometrium group regarding the age (*P* = 0.074), gestational age (*P* = 0.495), abdominal pain (*P* = 1), hemoglobin concentration (*P* = 0.872), and vaginal bleeding (*P* = 0.673), respectively. The EMT in women with repeated D&C (9.1 ± 3.1 mm; 95%CI: 5.2, 13) was lower than in women without D&C (16.3 ± 4.3 mm; 95%CI: 14.3, 18.2; *P* = 0.003; Fig. 4).

**Table 2:** MRI and clinical characteristics of patients with below-cutoff and above-cutoff endometrial thickness

Characteristics	Below-cutoff Endometrial Thickness (*n* = 6) ^a^	Above-cutoff Endometrial Thickness (*n* = 22) ^a^	*P* value
Age (year)	34.8 ± 4.2 (30.4, 39.2) ^b^	30.9 ± 4.5 (29.1, 32.7) ^b^	0.074 ^c^
Gestational Age (day)	49.7 ± 6.3 (43, 56) ^b^	52.3 ± 8.9 (48.7, 55.9) ^b^	0.495 ^c^
Abdominal Pain	0/6 (0)	3/22 (13.6%)	1 ^e^
Vaginal Bleeding	2/6 (33.3%)	10/22 (45.4%)	0.673 ^e^
Log ß-hCG (mIU/ml)	4.2 ± 0.6 (3.7, 4.8) ^b^	4.7 ± 0.4 (4.5, 4.8) ^b^	0.026 ^c^
Rate of repeated Dilatation & Curettage ^d^	4/6 (66.7%)	1/22 (4.6%)	0.003 ^e^
Peri-GS Endometrial Interruption	6/6 (100%)	2/22 (9.1%)	0.001^e^
Intraoperative blood Loss (ml)	28.3 ± 18.3 (13.6, 42.9) ^b^	10.1 ± 6.3 (7.3, 12.9) ^b^	0.001 ^c^
Hemoglobin Concentration(g/L)	126.3 ± 4.0 (122.2, 130.5) ^b^	125.5 ± 13.5 (111.3, 139.6) ^b^	0.872 ^c^
Hospital Stay (day)	7.5 ± 3.8 (4.5, 10.5) ^b^	2.8 ± 1.7 (2.1, 3.5) ^b^	0.001 ^c^

^a^ Cutoff value of endometrial thickness = 11.5 mm

^b^ Mean ± standard deviation (95% confidence interval)

^c^ Student’s *t* test

^d^ Two and more times of dilatation & curettage

^e^ Fisher’s exact test

GS: Gestational sac

### Association between MRI and clinical characteristics.

The incidence of peri-GS endometrial interruption was negatively associated with the EMT (OR, 0.55; 95% CI: 0.31, 0.78; *P* = 0.001) and logarithmic ß-hCG concentration (OR, 0.08; 95% CI: 0.008, 0.95; *P* = 0.045; Table 3). The below-cutoff EMT negatively correlated with logarithmic ß-hCG concentration (OR, 0.06; 95% CI: 0.002, 0.67; *P* = 0.021; Table 3).

**Table 3:** Associations between MRI and clinical characteristics

Dependent variables	Independent variables	OR	95% CI	***P*** value
**Below-cutoff endometrial thickness**	**Logarithmic ß-hCG**	0.06	0.002, 0.67	0.021
**Peri-GS endometrial interruption**	**Endometrial thickness**	0.55	0.31, 0.78	0.001
	**Logarithmic ß-hCG**	0.08	0.008, 0.95	0.045

OR: Odds ratio

95% CI: 95% Confidence interval

GS: Gestational sac

## Discussion

The pregnancy outcomes in women with surgery-categorized upper-lateral intracavitary pregnancy (ULIP) demonstrate higher heterogeneity than in women with ultrasonography-categorized ULIP. In this study, we elucidated that the surgery-categorized ULIP comprised two subtypes with either continuous (type-I) or interrupted peri-gestational sac (GS) endometrium (type-II). While the continuous peri-GS endometrium has been widely accepted as a key ultrasonographic feature to diagnose ULIP [3, 4], the type-II ULIP identified in the present study, with an interrupted peri-GS endometrium, hasn’t been reported in the literature. Additionally, the type-II ULIP, when compared to type-I, showed thinner endometrium, lower ß-hCG concentration, and a higher rate of dilation and curettage (D&C). The peri-GS endometrial interruption was negatively associated with the endometrial thickness (EMT) and ß-hCG concentration. The below-cutoff (11.5 mm) EMT negatively correlated with ß-hCG concentration.

A key MRI feature in women with type-II ULIP was the peri-GS endometrial interruption. An interrupted peri-GS endometrium indicates an incomplete endometrial surrounding of GS. A favorable pregnancy outcome starts from successful implantation, during which the endometrial stromal cells are highly reactive to the trophoblast-derived signals [9], resulting in an active engulfment of the blastocyst [10]. An endometrium-encapsulated embryo can be detected by ultrasonography in both centrally-implanted intrauterine pregnancy and ULIP, denoted as double sac [11] and fully surrounding endometrium [3], respectively. Having a fully endometrium-surrounded GS, the women with ultrasonography-categorized ULIP demonstrate a live birth rate of 80% [4]. In the present study, we showed that in addition to those with fully endometrium-surrounded GS, 21.4% (6/28) of the women with ULIP had an interrupted peri-GS endometrium associated with an EMT less than 11.5 mm and a higher D&C rate. The concurred peri-GS endometrial interruption and thinner endometrium in women with type-II ULIP suggest defective implantations that may result from impaired endometrial receptivity possibly caused by the repeated D&C [12–15].

The EMT in women with type-II ULIP was thinner than in women with type-I ULIP. EMT and endometrial volume post-implantation are reported to be positively correlated with favorable pregnancy outcomes [16–18]. During the first trimester, the thinner endometrium is associated with the occurrence of ectopic pregnancies [7, 19, 20]. Since we measured the EMT in the first trimester, literally a few weeks after natural conception, the thinner endometrium in women with type-II ULIP is more likely to suggest an unfavorable pregnancy outcome in these patients.

Women with type-II ULIP had a lower level of ß-hCG associated with thinner EMT. The endometrial expression of LH/hCG receptor implies that the ß-hCG may directly act on the endometrium to promote implantation, placentation, and decidualization in early pregnancy [21]. Exogenous hCG treatment induces a thicker endometrium than clomiphene or letrozole treatment [22]. Intrauterine ß-hCG facilitates the secretory transformation of the endometrium [23]. The negatively correlated below-cutoff EMT and ß-hCG concentration shown in our study imply that the ß-hCG may play a role in regulating the EMT during early pregnancy.

In type-II ULIP, the negatively associated peri-GS endometrial interruption and ß-hCG concentration may reflect a malfunctioning syncytiotrophoblast that is the primary source of ß-hCG after implantation [21]. Trophoblasts from early embryonic failure express significantly depressed levels of ß-hCG when compared to normal pregnancy [24]. Blood ß-hCG concentration in women with interstitial pregnancy is lower than in women with ULIP [7]. The lower level of ß-hCG in type-II ULIP suggests that the development of partially endometrium-encapsulated embryos in these patients might be defective. The below-cutoff EMT group demonstrated higher intraoperative blood loss and longer hospital stay during the procedure of deliberate termination, indicating that the women with type-II ULIP possess enhanced intraoperative and perioperative complexity [25, 26]. Therefore, in women with type-II ULIP, the mutually correlated thinner endometrium, peri-GS endometrial interruption, and lower ß-hCG concentration may act synergistically to increase the clinical complexity, which may eventually shift the pregnancy to various unfavorable outcomes, from early pregnancy loss to maternal death, documented in numerous published case reports [2].

Our study had limitations. First, this was a retrospective study with limited patient numbers. The bias-pro methodology and relatively low statistical power may impair the solidity of our results. Second, we lacked the follow-up data for each patient involved in this study owing to the deliberate termination undertaken after the MRI examination. Since the imaging-designated anatomical location of the gestational sac might be variable over time [3], the MRI features we identified may undergo further changes in the follow-up MRI scanning.

## Conclusions

We have elucidated that the surgery-categorized ULIP comprised two subtypes among which the type-II ULIP possesses unique imaging and clinical characteristics that haven’t been reported in the literature. Since the current ultrasonographic diagnostic criteria didn’t address the imaging features of the type-II ULIP identified in this study [3, 4], our results provided supplementary information to improve the accuracy of the imaging diagnosis for this obstetric condition. Well-designed prospective studies targeting both types of ULIP should be conducted to characterize a more comprehensive profile of the imaging features, clinical characteristics, pregnancy outcomes, and management strategies.

## Data Availability

The datasets generated or analyzed during the study are not publicly available due to the privacy protection policy of our institute but are available from the corresponding author on reasonable request.

## Abbreviations

ULIP: upper-lateral intracavitary pregnancy
EMT: endometrial thickness
GS: gestational sac
ß-hCG: ß-human chorionic gonadotropin
D&C: dilatation and curettage
